# Extracellular vesicles derived from *Staphylococcus aureus* induce atopic dermatitis-like skin inflammation

**DOI:** 10.1111/j.1398-9995.2010.02483.x

**Published:** 2011-03

**Authors:** S-W Hong, M-R Kim, E-Y Lee, J H Kim, Y-S Kim, S G Jeon, J-M Yang, B-J Lee, B-Y Pyun, Y S Gho, Y-K Kim

**Affiliations:** 1 Department of Life Science, Pohang University of Science and Technology (POSTECH)Pohang; 2 Department of Dermatology, Samsung Medical Center, Sungkyunkwan University School of MedicineSeoul, Korea; 3 Department of Allergy and Clinical Immunology, Samsung Medical Center, Sungkyunkwan University School of MedicineSeoul, Korea; 4 Department of Pediatrics, Suncheonhyang University College of MedicineSeoul, Korea

**Keywords:** atopic dermatitis, extracellular vesicles, skin inflammation, *Staphylococcus aureus*

## Abstract

**Background:**

Recently, we found that *Staphylococcus aureus* produces extracellular vesicles (EV) that contain pathogenic proteins. Although *S. aureus* infection has been linked with atopic dermatitis (AD), the identities of the causative agents from *S. aureus* are controversial. We evaluated whether *S. aureus*-derived EV are causally related to the pathogenesis of AD.

**Methods:**

Extracellular vesicles were isolated by the ultracentrifugation of *S. aureus* culture media. The EV were applied three times per week to tape-stripped mouse skin. Inflammation and immune dysfunction were evaluated 48 h after the final application in hairless mice. Extracellular vesicles-specific IgE levels were measured by ELISA in AD patients and healthy subjects.

**Results:**

The *in vitro* application of *S. aureus* EV increased the production of pro-inflammatory mediators (IL-6, thymic stromal lymphopoietin, macrophage inflammatory protein-1α, and eotaxin) by dermal fibroblasts. The *in vivo* application of *S. aureus* EV after tape stripping caused epidermal thickening with infiltration of the dermis by mast cells and eosinophils in mice. These changes were associated with the enhanced cutaneous production of IL-4, IL-5, IFN-γ, and IL-17. Interestingly, the serum levels of *S. aureus* EV-specific IgE were significantly increased in AD patients relative to healthy subjects.

**Conclusion:**

These results indicate that *S. aureus* EV induce AD-like inflammation in the skin and that *S. aureus*-derived EV are a novel diagnostic and therapeutic target for the control of AD.

Skin lesions in atopic dermatitis (AD) patients display histological alterations such as epidermal thickening and infiltration by eosinophils and mast cells (1). The pathogenesis of AD is believed to involve repeated abnormal innate and adaptive immune responses to environmental causative agents when the skin barrier is disrupted (2). Aeroallergens, including house dust mites and pollens, are well known to induce AD through IgE-mediated mechanisms (3). In addition, microbes represent another important group of extrinsic causative factors in the pathogenesis of AD (4). *Staphylococcus aureus* (*S. aureus*) appears to be particularly important because it colonizes almost all lesional skin in AD patients, and a reduction in its colonization has been shown to decrease disease severity (5–7). Some AD patients produce IgE antibodies specific to staphylococcal enterotoxin A or B (SEA or SEB) (8, 9). Lipoteichoic acid, cell wall component in *S. aureus*, has been detected in the lesions of AD patients and is correlated with AD severity (10). This finding together with the fact that about 20–40% of AD patients display intrinsic AD with no sensitization to any protein allergen suggests the importance of microbes (11).

Gram-negative bacteria secrete outer membrane vesicles (OMV) (12), which have pathogenic effects (13, 14). Recently, we demonstrated for the first time that the Gram-positive bacterium *S. aureus* produces OMV-like vesicles called extracellular vesicles (EV) (15). The EV produced by *S. aureus* are spherical with a diameter of 20–100 nm and are shed from the bacterium's membranes. Proteomic analysis revealed that the protein expression pattern in these EV differs from that in whole bacteria, and that they contain various pathogenic molecules. Among these, α-hemolysin and cysteine protease have been linked with AD (16, 17). These findings suggest that EV is a potent initiator of host immune responses.

In AD patients, *S. aureus*, rather than invading and infecting the skin, colonizes it. It is thus reasonable to assume that the skin is affected by secretory products from *S. aureus*. Given that staphylococcal secretory products are relevant to the pathogenesis of AD, we hypothesized that *S. aureus* EV, which are complexes of various pathogenic molecules secreted by the bacterium, are involved in the pathogenesis of AD. In this study, we found through *in vitro* and *in vivo* studies that *S. aureus* EV are causative agents in AD, and the clinical observation also supports this hypothesis.

## Methods

### Mice

SKH-HR1 (hairless) mice were purchased from Charles River Laboratories Japan, Inc. (Yokohama, Japan) and were bred in a pathogen-free facility at Pohang University of Science and Technology (POSTECH; Pohang, Korea). All animal experiments were approved by the POSTECH Ethics Committee.

### Patients

Skin lavage fluids were obtained from two AD patients visiting Pediatric Clinic of Seoul Suncheonhyang Hospital (Seoul, Korea). Serum samples were obtained from 60 AD patients (30 patients aged 6–9 years and 30 patients aged over 10 years) and 20 healthy subjects aged 6–16 years, who were recruited from Seoul Samsung Hospital (Seoul, Korea). Skin lavage fluids and serum samples were isolated after written informed consent had been obtained. The study protocol was approved by the Ethics Committee of Seoul Suncheonhyang Hospital and Seoul Samsung Hospital, respectively.

### Isolation of *Staphylococcus aureus* extracellular vesicles

*Staphylococcus aureus* EV were obtained as described previously (15). Briefly, *S. aureus* (ATCC14458) was cultured in nutrient broth (Merck, Darmstadt, Germany) and grown at 37°C to 1.0 of optical density (at 600 nm). Bacteria were removed by centrifugation, and the resulting supernatant was filtered through a 0.45-μm vacuum filter. The filtrate was concentrated by ultrafiltration using the QuixStand Benchtop System (Amersham Biosciences, Piscataway, NJ, USA) in conjunction with a 100-kD hollow-fiber membrane (Amersham Biosciences). The resulting concentrated filtrate was passed through a 0.22-μm vacuum filter. Extracellular vesicles were isolated from the resulting filtrate by ultracentrifugation at 150 000 ***g***. The concentration of protein in the EV was measured by Bradford assays (Bio-Rad Laboratories, Hercules, CA, USA). Hereafter, the reported doses of EV refer to the amount of EV protein. Isolated EV were stored at −80°C before use. Bacteria and EV removed, and then < 100 and >100-kD soluble fractions of bacterial culture media were concentrated. Protein concentration was measured by Bradford assays, and culture media were stored at −80°C before use.

### Detection of staphylococcal enterotoxin A and staphylococcal enterotoxin B

The presence of SEA and SEB in EV and concentrated culture media was analyzed using SDS-PAGE and western blot. SEA and SEB were detected by monoclonal anti-SEA and anti-SEB antibody (Santa Cruz Biotechnology, Santa Cruz, CA, USA).

### The generation of an atopic dermatitis mouse model using *Staphylococcus aureus* extracellular vesicles

To create a mouse model of AD, *S. aureus* EV were applied to mouse skin according to the following protocol. To disrupt the cutaneous barrier, the dorsal skin of 6-week-old mice was stripped five to six times using Durapore surgical tape (3M Co., St. Paul, MN, USA). Gauze (1.5 × 1.5 cm) soaked with *S. aureus* EV in 100 μl of phosphate buffered saline (PBS) was then placed on the stripped skin and secured using Tegaderm bio-occlusive tape (3M Co.). For the evaluation of inflammation and immune dysfunction, the mice were euthanized 48 h after the final challenge.

### Histological analysis

Four-micrometer-thick sections of fixed skin tissues were stained with hematoxylin and eosin (H&E). Mast cells were stained with toluidine blue (TB). Cells were counted in 15–25 high-power fields at a magnification of ×400.

### Characterization of T-cell subsets

Single cells of skin-draining lymph nodes (LN) were collected and stimulated with or without 0.1 μg/ml of *S. aureus* EV. Supernatants were harvested after 72 h, and the levels of cytokines measured by ELISA.

### *In vitro* production of pro-inflammatory mediators from dermal fibroblasts

Primary mouse dermal fibroblasts were isolated as described previously with some modification (18). Fibroblasts from passages 1–3 were used. Then, 2 × 10^4^ cells of isolated cells were cultured in 24-well plates then treated with 1 or 10 μg/ml *S. aureus* EV or soluble fractions of bacterial culture media, or SEB (Toxin Technology, Sarasota, FL, USA). Supernatants were collected 24 h after stimulation, and mediator levels measured.

### Measurement of cytokine and chemokine secretion

The cytokine and chemokine levels were measured by ELISA (R&D Systems, Mineapolis, MN, USA) according to the manufacturer's instructions.

### Isolation of extracellular vesicles from the skin lavage fluids of patients

Skin lavage fluids were obtained by rinsing patients' skin lesions three to four times with 50 ml of sterile PBS and were stored at −80°C. To remove bacteria and other debris, 40 ml of skin lavage fluids was centrifuged at 5000 and 10 000 ***g***. After centrifugation, supernatants were filtered through 0.45 and 0.22 μm serially. Then, lavage fluids were concentrated to 1 ml by using Centriprep YM-50 (Millipore, Carringtwohill, Ireland). Same volume of PBS was added on concentrated lavage fluids, and they were ultracentrifuged at 150 000 ***g*** for 3 h at 4°C. The pellet was used as EV fraction.

### ELISA assay using anti-*Staphylococcus aureus* extracellular vesicles-specific polyclonal antibodies

Anti-*S. aureus* EV-specific polyclonal antibodies were coated on 96-well ELISA plate. Each well was blocked by 1% bovine serum albumin in PBS. After blocking, concentrated lavage fluids and EV fraction were added to each well. Then, biotinylated anti-*S. aureus* EV-specific polyclonal antibodies were added on each well, and then streptavidin-horseradish peroxidase (HRP) was added. After a final wash, chemiluminescence substrates (POD) were added to react with HRP. Luminescence measured using a Wallac 1420 Victor luminometer (American Instrument Exchange, Inc., Haverville, MA, USA).

### Serum antibody ELISA

#### Mouse

Serum samples were prepared from mouse blood for analysis of the total serum IgG1 and IgE levels by ELISA (Bethyl Laboratories, Montgomery, TX, USA) according to manufacturer's instructions.

#### Human

*Staphylococcus aureus* EV- and SEB-specific IgG1 and IgE levels were measured by ELISA. The wells of 96-well ELISA plates were each coated with 0.1 μg of *S. aureus* EV or 1 μg of SEB. Then, wells were blocked with 3% bovine serum albumin in PBS. After blocking, diluted human serum was added to the wells. Then, HRP-conjugated anti-human IgG1 and IgE antibodies (Southern Biotech, Birmingham, IL, USA) were applied to each well. Chemiluminescence substrates were added, and luminescence was measured by the luminometer. Specific antibody levels were defined as elevated if a value is higher than mean +1 standard deviation of healthy subjects' values.

### Statistical analysis

Statistically significant differences between treatments were identified using Student's *t*-test, anova, or Wilcoxon's rank sum test. Multiple comparisons were initially made by anova. Where significant differences were found, individual *t*-tests or Wilcoxon's rank sum tests were used to identify statistically significant differences between treatment group pairs. A *P*-value < 0.05 was considered to be statistically significant.

## Results

### *In vitro* production of pro-inflammatory mediators from mouse dermal fibroblasts treated with *Staphylococcus aureus* extracellular vesicles

**Figure 1:**
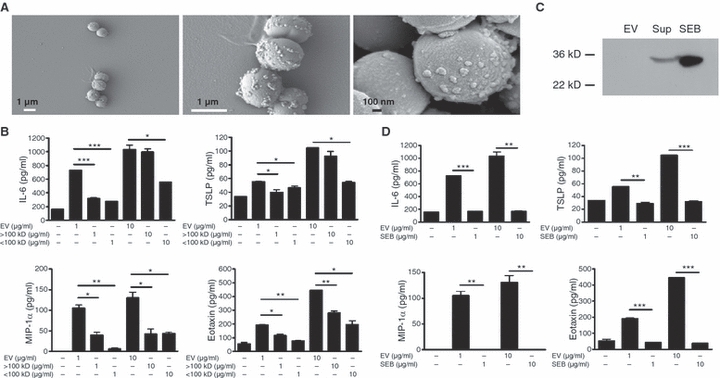
*Staphylococcus aureus* extracellular vesicles (EV) enhance the *in vitro* secretion of immune and pro-inflammatory mediators by mouse dermal fibroblasts. (A) Scanning electron microscopic images that *S. aureus* secrete EV. Arrowheads identify *S. aureus* EV. (B) Levels of pro-inflammatory mediators IL-6, thymic stromal lymphopoietin, macrophage inflammatory protein-1α and eotaxin in supernatants of dermal fibroblasts after stimulation with EV and >100- and < 100-kD soluble fractions of bacterial culture media. (C) Western blotting to detect staphylococcal enterotoxin B (SEB) in EV and soluble (Sup) fractions of *S. aureus* culture media. (D) Levels of pro-inflammatory mediators from supernatants of dermal fibroblasts after stimulation with EV or SEB. Assays were performed in duplicate. *P < 0.05; **P < 0.01; ***P < 0.001.

Recently, we found that *S. aureus* EV contain 90 proteins, including proteins with pathological function, by proteomic analysis (15). Scanning electron microscopic images showed that *S. aureus* secreted EV (Fig. 1A). We evaluated whether EV or soluble fractions of *S. aureus* culture media induce the production of pro-inflammatory mediators from skin fibroblasts. We found that the production of IL-6, thymic stromal lymphopoietin (TSLP), macrophage inflammatory protein (MIP)-1α, and eotaxin by dermal fibroblasts was higher by stimulation with EV than with soluble fraction (Fig. 1B). It was reported that the presence of IgE antibodies to SEA and SEB was correlated with the severity of skin lesions in children with AD (8, 9). Western blotting using anti-SEA and anti-SEB antibodies showed that SEB was present in soluble fraction, but not in EV, whereas SEA absent in both fractions (Fig. 1C). We compared *in vitro* activity between EV and SEB on the production of pro-inflammatory mediator. As shown in Fig. 1D, 1 or 10 μg/ml of SEB did not enhance the production of IL-6, TSLP, MIP-1α, and eotaxin, whereas 1 μg/ml of EV upregulated the production of these mediators. Collectively, these findings suggest that *S. aureus*-derived EV are more potent compared to soluble components, in terms of production of pro-inflammatory mediators.

### Local inflammation induced by the application of different doses of *Staphylococcus aureus* extracellular vesicles to tape-stripped mouse skin

**Figure 2:**
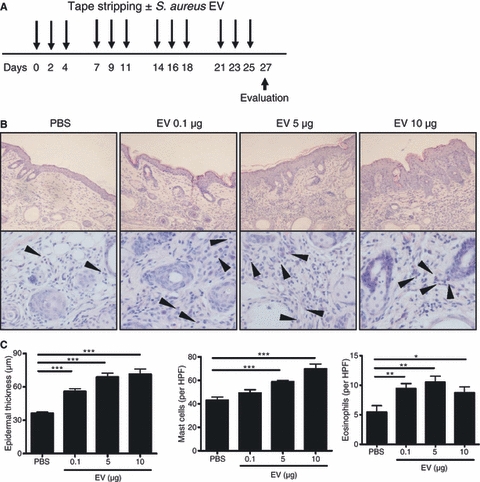
Application of *Staphylococcus aureus* extracellular vesicles (EV) to tape-stripped mouse skin induces atopic dermatitis-like inflammation. (A) Study protocol: application of different doses of *S. aureus* EV to tape-stripped mouse skin for 4 weeks (*n* = 5 per treatment group). (B) Skin histology [H&E staining; magnification, ×200 (upper panel) and ×400 (lower panel)]. Arrowheads identify eosinophils. (C) Histological analysis of epidermal thickness and the numbers of eosinophils and mast cells infiltrating the dermis. *P < 0.05; **P < 0.01; ***P < 0.001.

We evaluated the *in vivo* effects of *S. aureus* EV on the induction of skin inflammation. Different doses of EV were applied to tape-stripped mouse skin, and changes in skin inflammation were assessed 4 weeks after initial application (Fig. 2A). Histological analysis showed that the application of *S. aureus* EV to tape-stripped skin induced AD-like inflammation, including epidermal thickening and infiltration of the dermis by inflammatory cells (Fig. 2B). Moreover, *S. aureus* EV dose dependently induced epidermal thickening (Fig. 2C). As with dermal infiltration by inflammatory cells, significantly higher numbers of mast cells were found in the dermis of mice treated with *S. aureus* EV (at 5 or 10 μg, but not 0.1 μg) compared to PBS-treated controls. In addition, the numbers of eosinophils were significantly higher in EV-treated mice than in PBS-treated controls at all doses (Fig. 2C). Together, these data suggest that the application of *S. aureus* EV to tape-stripped skin induces AD-like inflammation.

### *In vivo* immune dysfunction induced by the application of *Staphylococcus aureus* extracellular vesicles to tape-stripped mouse skin for 3 weeks

**Figure 3:**
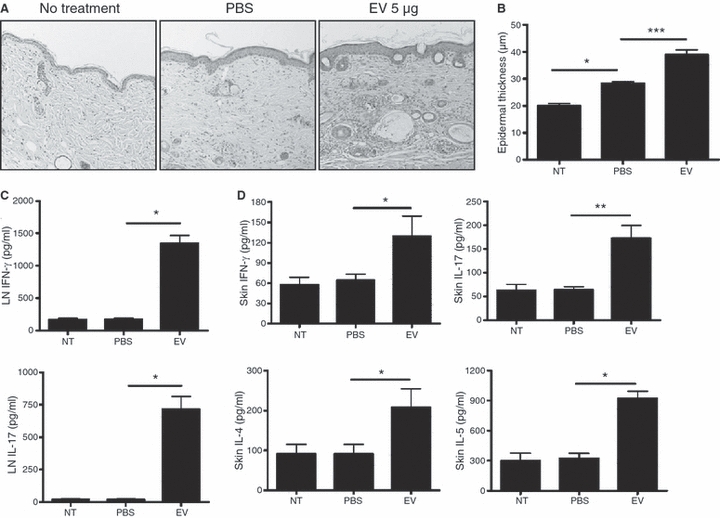
Three-week exposure of tape-stripped mouse skin to *Staphylococcus aureus* extracellular vesicles (EV) induces a mixed Th1-/Th17-/Th2-type inflammatory response in the skin, and the generation of Th1 and Th17 cells in skin-draining lymph nodes (LNs). Evaluation (*n* = 5 per treatment group) was performed 48 h after the final application of *S. aureus* EV (5 μg) (performed three times a week for 3 weeks) to tape-stripped skin. *P < 0.05; **P < 0.01; ***P < 0.001. (A) Skin histology [H&E staining; magnification, ×200]. (B) Histological analysis of epidermal thickness. (C) Levels of IFN-γ and IL-17 in supernatants from *S. aureus* EV-treated cells from skin-draining LNs. (D) Levels of IFN-γ, IL-17, IL-4, and IL-5 in skin tissue homogenates.

To test whether the application of *S. aureus* EV to the skin induces immunological dysfunction, 5 μg of *S. aureus* EV was applied to tape-stripped mouse skin three times per week for 3 weeks. Histological analysis showed that the application of *S. aureus* EV induced epidermal thickening and infiltration of the dermis by inflammatory cells (Fig. 3A). Tape stripping itself induced epidermal thickening (Fig. 3B). To characterize the immune dysfunction induced by *S. aureus* EV, we measured the production of Th1, Th17, and Th2 cytokines by T cells from skin-draining LNs following *in vitro* stimulation with *S. aureus* EV. The production of IFN-γ and IL-17 was significantly higher in cells from the EV-treated mice than in those from the PBS-treated animals (Fig. 3C). IL-4 and IL-5 were not detected in the supernatants from cells isolated from either treatment group (data not shown). In terms of the skin production of Th1, Th17, and Th2 cytokines, the levels of IFN-γ, IL-17, IL-4, and IL-5 in skin homogenates from EV-treated mice were significantly higher than those from PBS-treated mice. Tape stripping did not itself enhance the production of these cytokines (Fig. 3D). With regard to the production of antibodies, we found the serum levels of total IgG1 and IgE to be similar in the EV- and PBS-treated mice (data not shown). Collectively, these data suggest that exposure to *S. aureus* EV for 3 weeks induces a mixed Th1-/Th17-/Th2-type inflammatory response in the skin, whereas a mixed Th1/Th17 cell response in skin-draining LNs.

### Local inflammation and systemic immune dysfunction induced by long-term (8-week) application of *Staphylococcus aureus* extracellular vesicles to tape-stripped mouse skin

**Figure 4:**
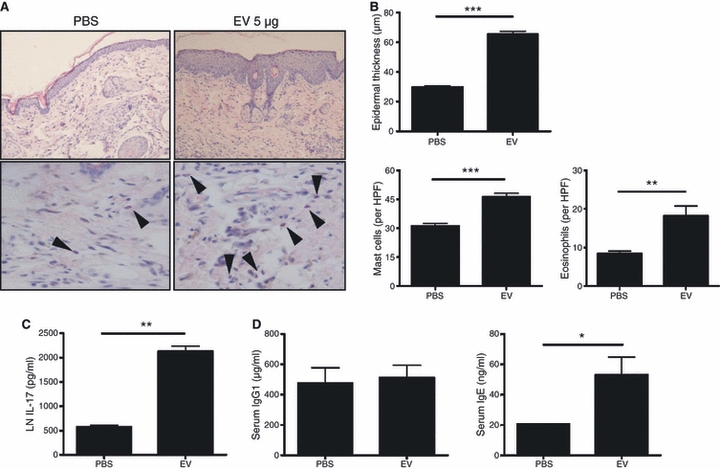
Long-term exposure of tape-stripped mouse skin to *Staphylococcus aureus* extracellular vesicles (EV) enhances the production of IgE. Evaluation (*n* = 5 per treatment group) was performed 48 h after the final application of *S. aureus* EV (5 μg) (performed three times a week for 8 weeks) to tape-stripped skin. *P < 0.05; **P < 0.01; ***P < 0.001. (A) Skin histology [H&E staining; magnification, ×200 (upper panel) and ×400 (lower panel)]. Arrowheads identify eosinophils. (B) Histological analysis of epidermal thickness and numbers of eosinophils and mast cells infiltrating the dermis. (C) Levels of IL-17 in supernatants from *S. aureus* EV-treated cells from skin-draining lymph nodes. (D) Serum levels of total IgG1 and total IgE.

To assess the effects of long-term exposure to *S. aureus* EV, *S. aureus* EV (5 μg) were applied to tape-stripped skin three times per week for 8 weeks. Histological analysis showed that prolonged exposure to *S. aureus* EV induced epidermal thickening and increased infiltration of the dermis by inflammatory cells (Fig. 4A,B). Infiltration of the dermis by mast cells and eosinophils was significantly increased in EV-treated mice relative to PBS-treated controls (Fig. 4A,B). Furthermore, long-term *in vivo* exposure to *S. aureus* EV enhanced the production of IL-17 by T cells in skin-draining LNs stimulated with *S. aureus* EV *in vitro* (Fig. 4C). However, it did not enhance the EV-induced production of IFN-γ and IL-4 (data not shown). In terms of antibody production following long-term exposure to *S. aureus* EV, the serum total IgE levels were significantly higher in the EV-treated mice than in the PBS-treated controls, although the serum total IgG1 levels were similar between the two groups (Fig. 4D). Taken together, these findings suggest that long-term exposure to *S. aureus* EV induces IgE production, whereas Th17-cell response in skin-draining LNs.

### The presence of *Staphylococcus aureus* extracellular vesicles in the skin lesion of atopic dermatitis patients

**Figure 5:**
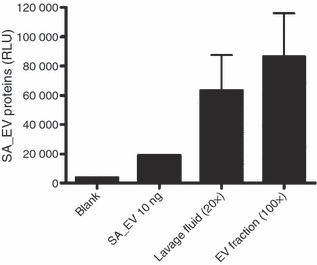
*Staphylococcus aureus*-derived extracellular vesicles (EV) are present on the skin of atopic dermatitis (AD) patients. This figure showed ELISA assay to detect *S. aureus* EV-specific proteins using anti-*S. aureus* EV polyclonal antibodies; lavage fluids and EV fraction of lavage fluids obtained from two AD patients have *S. aureus* EV-specific proteins (SA_EV, *S. aureus*-derived EV).

We evaluated the presence of *S. aureus*-derived EV in the skin lesion of AD patients. To test this objective, EV were isolated from the skin lesion of two AD patients and then evaluated whether EV isolated from the skin lesion of AD patients incorporate *S. aureus* EV-specific proteins using anti-*S. aureus* EV-specific polyclonal antibodies. As shown in Fig. 5, EV isolated from skin lavage fluids of AD patients contained *S. aureus* EV-specific proteins.

### Serum *Staphylococcus aureus* extracellular vesicles-specific antibody levels in atopic dermatitis patients

**Figure 6:**
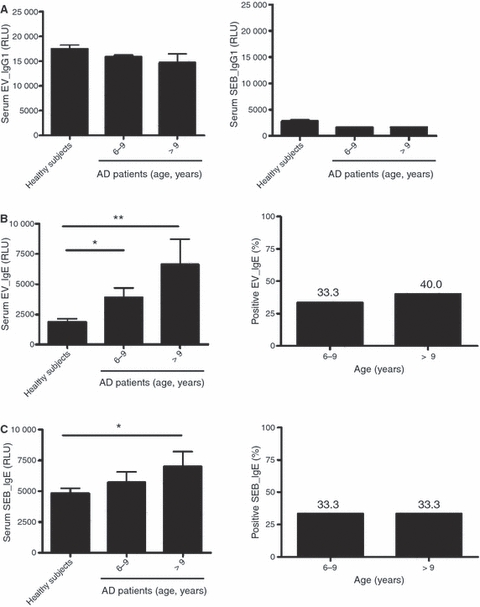
*Staphylococcus aureus* extracellular vesicles (EV)-specific IgE is elevated in atopic dermatitis (AD) patients than in age-matched healthy subjects. (A) Levels of *S. aureus* EV-specific (left panel) and SEB-specific (right panel) IgG1 in serum from AD patients and healthy subjects. (B) Levels of *S. aureus* EV-specific IgE in serum from AD patients and healthy subjects (left panel), and positive rate of elevated *S. aureus* EV-specific IgE in AD patients (right panel). (C) Levels of SEB-specific IgE in serum from AD patients and healthy subjects (left panel), and positive rate of elevated SEB-specific IgE in AD patients (right panel). Serum samples from AD patients (*n* = 30 in patients aged 6–9 years, and *n* = 30 in patients aged 9–16 years) and healthy subjects aged 6–16 years (*n* = 20); EV, *S. aureus*-derived EV; SEB, staphylococcal enterotoxin B. *P < 0.05; **P < 0.01.

Finally, we measured serum levels of *S. aureus* EV- and SEB-specific antibodies in AD patients and healthy subjects. The serum levels of *S. aureus* EV- and SEB-specific IgG1 were comparable in AD patients and healthy subjects (Fig. 6A). By contrast, the serum *S. aureus* EV-specific IgE levels were significantly higher in both 6–9 year aged and >9 year aged AD patients than in age-matched healthy subjects; this IgE levels were elevated in 33.3% of 6–9 years aged and 40% of >9 years aged AD patients (Fig. 6B). In addition, the serum SEB-specific IgE levels were significantly higher in only >9 years aged AD patients than in age-matched healthy subjects; this IgE levels were elevated in 33.3% of 6–9 years aged and 33.3% of >9 years aged AD patients (Fig. 6C). However, neither total IgE nor SEB-specific IgE were correlated with *S. aureus* EV-specific IgE in AD patients (data not shown). These findings indicate that *S. aureus* EV-specific IgE may be a useful biomarker for identifying the cause of AD in individual cases.

## Discussion

Elucidating the pathogenesis of AD has been difficult because of complex interactions between the causative factors and host immune response. Among the known causative factors, microbes are thought to be particularly important in the pathogenesis of AD by elaborating secretary products, including soluble toxins. Recently, we found that *S. aureus* secretes EV, in which pathogenic proteins are incorporated (15). In the present study, experimental and clinical data support the hypothesis that *S. aureus* EV are involved in the pathogenesis of AD.

In terms of host immune responses of AD pathogenesis, recent studies showed that IL-17 producing cells are found in the blood of AD patients and that AD pathogenesis is related with IL-17-mediated immune responses (19, 20). In the present study, we found that the cutaneous application of *S. aureus* EV induced Th17-cell response in skin-draining LNs, suggesting that Th17-cell response is important in the AD pathogenesis.

The present data showed that the cutaneous application of *S. aureus* EV induced skin inflammation characterized by infiltration of mast cells and eosinophils. This inflammatory response was associated with enhanced production of not only Th1/Th17-type cytokines, but also Th2-type cytokines in the skin. In addition, the present study showed that *in vitro* stimulation of fibroblasts with *S. aureus* EV increased the secretion of the Th2-type cytokines, such as TSLP and eotaxin (21). These findings suggest that Th2-type inflammation induced by *S. aureus* EV is mediated by local production of Th2-type cytokines from dermal fibroblasts.

*Staphylococcus aureus* can colonize in skin or nasal passage in human (22). In the present study, we firstly demonstrated that *S. aureus*-derived EV are present on the skin of AD patients. We also found that *S. aureus* EV-specific IgG1 were detected in serums from not only AD patients but also healthy subjects. These findings suggest that *S. aureus* secretes EV in skin, which induce systemic immune responses.

Previous studies showed that serum levels of IgE specific to staphylococcal enterotoxins were not only elevated in AD patients, but also correlated with disease severity (8, 9). Our present data indicate that serum levels of both *S. aureus* EV- and SEB-specific IgE were elevated in the AD patients compared to healthy subjects. In addition, the present study showed that *S. aureus* EV did not contain enterotoxins and *S. aureus* EV-specific IgE levels did not correlate with SEB-specific IgE. These findings suggest that the production of EV-specific IgE is not influenced by staphylococcal enterotoxins.

Recently, it was reported that IL-17 can induce IgE production in B cells (23). The present study showed that the cutaneous application of *S. aureus* EV for 8 weeks induce Th17-cell response, but not Th2-cell response, along with the elevation of serum total IgE in mice. These findings suggest that *S. aureus* EV can induce systemic IgE production by Th17-cell response.

Recent evidence indicates that EV from Gram-negative bacteria induced systemic inflammatory response (13). We found that Gram-positive bacteria produced EV (15). This is the first report to show that EV obtained from Gram-positive bacteria can cause inflammatory disease. Given the abundance of Gram-positive bacteria in our environment, further research will be needed to elucidate the relationships between EV from Gram-positive bacteria and the pathogenesis of immune-based inflammatory diseases.

In summary, our present data indicate that *S. aureus*-derived EV can induce AD-like inflammation in the skin, and that the physiological animal model we employed represents a useful tool for performing translational research into AD. Furthermore, our clinical findings provide strong evidence of the importance of *S. aureus* EV in the pathogenesis of AD.
